# Sonoproduction of nanobiomaterials – A critical review

**DOI:** 10.1016/j.ultsonch.2021.105887

**Published:** 2021-12-22

**Authors:** Sze Shin Low, Maxine Yew, Chang Nong Lim, Wai Siong Chai, Liang Ee Low, Sivakumar Manickam, Beng Ti Tey, Pau Loke Show

**Affiliations:** a Research Centre of Life Science and Healthcare, China Beacons Institute, University of Nottingham Ningbo China, 199 Taikang East Road, Ningbo 315100, Zhejiang, China; b Department of Mechanical, Materials and Manufacturing Engineering, University of Nottingham Ningbo China, 199 Taikang East Road, Ningbo 315100, Zhejiang, China; c School of Engineering and Physical Sciences, Heriot-Watt University Malaysia, No. 1, Jalan Venna P5/2, Precinct 5, Putrajaya 62200, Malaysia; d School of Mechanical Engineering and Automation, Harbin Institute of Technology, Shenzhen 518055, Guangdong, China; e Biofunctional Molecule Exploratory (BMEX) Research Group, School of Pharmacy, Monash University Malaysia, Jalan Lagoon Selatan, Bandar Sunway 47500, Selangor Darul Ehsan, Malaysia; f Advanced Engineering Platform, Monash University Malaysia, Jalan Lagoon Selatan, Bandar Sunway 47500, Selangor Darul Ehsan, Malaysia; g Chemical Engineering Discipline, School of Engineering, Monash University Malaysia, Jalan Lagoon Selatan, Bandar Sunway 47500, Selangor Darul Ehsan, Malaysia; h Petroleum and Chemical Engineering, Faculty of Engineering, Universiti Teknologi Brunei, Jalan Tungku Link Gadong, Bandar Seri Begawan BE1410, Brunei Darussalam; i Department of Chemical and Environmental Engineering, Faculty of Science and Engineering, University of Nottingham Malaysia, Jalan Broga, Semenyih 43500, Selangor Darul Ehsan, Malaysia

**Keywords:** Ultrasound, Cavitation, Nano, Material, Nanobiomaterial, Synthesis, Sonoproduction

## Abstract

Ultrasound (US) demonstrates remarkable potential in synthesising nanomaterials, particularly nanobiomaterials targeted towards biomedical applications. This review briefly introduces existing top-down and bottom-up approaches for nanomaterials synthesis and their corresponding synthesis mechanisms, followed by the expounding of US-driven nanomaterials synthesis. Subsequently, the pros and cons of sono-nanotechnology and its advances in the synthesis of nanobiomaterials are drawn based on recent works. US-synthesised nanobiomaterials have improved properties and performance over conventional synthesis methods and most essentially eliminate the need for harsh and expensive chemicals. The sonoproduction of different classes and types of nanobiomaterials such as metal and superparamagnetic nanoparticles (NPs), lipid- and carbohydrate-based NPs, protein microspheres, microgels and other nanocomposites are broadly categorised based on the physical and/or chemical effects induced by US. This review ends on a good note and recognises US-driven synthesis as a pragmatic solution to satisfy the growing demand for nanobiomaterials, nonetheless some technical challenges are highlighted.

## Highlights

• Ultrasound (US) is a powerful synthesis method for a multitude of nanobiomaterials.
• US-synthesised nanobiomaterials display better properties and performance.
• Top-down and bottom-up nanobiomaterials synthesis are evaluated.
• Mechanisms involved in the synthesis process are discussed.
• Main challenges of US-synthesised nanobiomaterials are addressed.

## Graphical abstract

**Figure:**
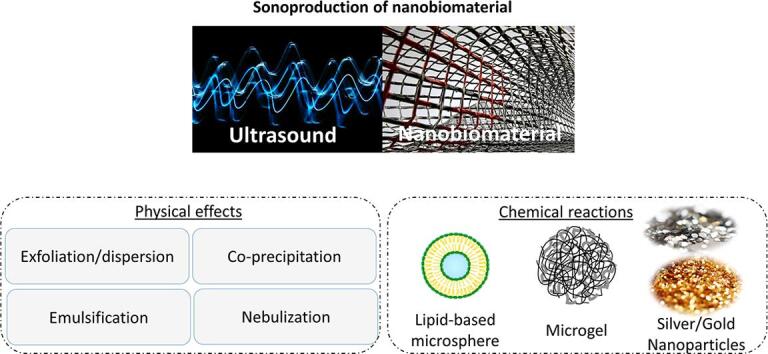

## 1 Introduction

Nanobiomaterials are used in biomedical applications, and their development has been progressing rapidly over the past decade [1]. Recent advancements in nanotechnology pave exciting opportunities for developing advanced nanobiomaterials for medical treatments using ultrasound (US). Nanobiomaterials could be applied in a wide range of biomedical fields, including diagnostics, drug delivery and treatment [2], [3], [4]. The low toxicity and antimicrobial effect of nanobiomaterials are particularly useful against the host, as well as its specific targets [5]. Despite numerous works on the production of nanobiomaterials, their synthesis process still requires harsh chemicals and are not environmentally friendly [6], [7].

The application of powerful high-intensity US radiation to chemical reactions called “sonochemistry” is utilised to produce nanostructured biomaterials [8]. US is favourable to apply in material synthesis and has always been coupled with other methods to achieve a synergistic effect that improves the overall synthesis process [9]. The ultrasonic radiation is capable of mixing and heating the precursor to the concentrated tiny hot energy spots that are intense enough to produce high-energy chemical reactions, thereby synthesising nanomaterials without the need for high temperatures, pressures, or long reaction times that are usually required in conventional synthesis approaches [10]. The acoustic cavitation for the physical and chemical effects of the sonochemical process involves three distinct stages: formation, growth, and implosive collapse of bubbles [11]. Employing the US technique is especially useful in synthesising hollow micro- or nano- spherical and other novel materials. This is due to the complex and wide range of processes induced by US, leading to nanobiomaterials with various structures and modifications tailor-made for drug delivery and diagnostics applications.

Extreme conditions (5000 K and 1000 bar) can be achieved within a short timeframe, which drives the chemical reactions and results in the obtainment of nanomaterials [7]. US-synthesised nanobiomaterials have the advantages of controlled structure and dimension by changing the synthesis parameters. Compared to conventional synthesis methods, US-synthesis is more environmentally friendly and consumes only a fraction of the energy [12], [13]. US-assisted synthesis improves the quality and yield of graphene layers [14]. Recent work has also examined the usage of US for drug synthesis and delivery purposes [15], [16], [17].

The scope of US-assisted synthesis of nanoparticles (NPs) is vast, as studies of NPs across different fields have surged in recent years [4]. With the breakthrough and extensive use of NPs in biomedical applications, the surge in demand necessitates a more effective means of producing high-quality NPs, which is the key constraint for the synthesis of NPs [18], [19]. Hence the focus of this review is mainly on US-assisted nanobiomaterials synthesis for various biomedical applications. Different mechanisms in the US-assisted nanobiomaterial synthesis are discussed, and various nanobiomaterials synthesis strategies are also evaluated. The sonoproduction of various nanobiomaterials (lipid, carbohydrate, hydrogel, metal and others) are assessed. The main challenges of US-synthesised nanobiomaterials have also been prospected.

## 2 Production of nanomaterials using US

Following the discovery of US by the Italian physicist Lazaro Spallanzani in 1794, when analysing the navigation mechanism of the flying bats, the Curies in 1878–1880 discovered the piezoelectric effect [20], [21], which is the capability to create electricity by mechanical vibrations induced by the US on quartz crystal, which became the basis of producing US from electrical signal. Sound waves are longitudinal pressure waves generated from the vibration of particles that propagate through the transmission medium, such as solid, liquid or gas. The frequency of the sound waves is correlated to acoustic velocity and wavelengths combined to form a high-pressure region, known as compression, and subsequent separation to form a low-pressure region, known as rarefaction. The distance between two areas of compression or rarefaction is known as wavelength. Wavelengths are shorter at high frequency leading to low penetration due to absorption and attenuation whilst at lower frequency strictly below 2 MHz, great acoustic energy induces cavitation in liquid medium [22].

The advantages of US have been extensively studied in the production of nanomaterials, as demonstrated in the following sections. Ultrasonic irradiation can induce high-energy chemical reactions in liquids leading to sonochemistry and sonoluminescence, the basic mechanism of which arises from acoustic cavitation: formation (nucleation), growth (expansion), and implosive collapse of bubbles [23]. The implosion of cavitation bubbles produces intense local heating (5000 K) and high pressures (20 MPa) with short lifetimes, resulting in extreme heating and cooling rates above 10^10^ K/s [24]. This creates a high-energy micro-reactor environment leading to extremely high energy chemical reaction within the vapour phase in the cavitation bubble and the fluid surrounding it [25], [26], [27]. The hot-spot theory explains the mechanism of sonochemistry and sonoluminescence; potential energy given to the bubble during its maximum expansion is concentrated into a heated gas core during bubble implosion.

### 2.1 Synthesis of nanomaterials

Nanomaterials possess different chemical and physical properties due to their size, surface, and interface effects than their bulk counterparts. They can be defined as materials with at least one dimension in the nanometre scale (10^-9^ m). Intense research is directed towards nanomaterials and has become an active area since discovering the potential in nanoscience and nanotechnology leading to the great advancement of nanoscale investigations across all scientific disciplines such as catalysis, controlled release systems and biotechnologies. Nanomaterials can be synthesised by various approaches in achieving different sizes, shapes, structures, and crystallinity [28], [29], [30], [31], [32], [33]. This section briefly describes the common methods to produce nanomaterials.

The strategies developed to obtain nanomaterials can be classified into top-down and bottom-up. The top-down approach reduces bulk material’s size to nanometric scale via selective removal or destruction such as mechanical milling, photolithography, laser ablation, chemical etching, and thermal decomposition. Mechanical milling is a low-cost and effective method, especially in producing nanocomposites such as oxide- and carbide-strengthened aluminium alloys and wear-resistant spray coatings [34]. It has been used extensively for milling and post-annealing of NPs during synthesis. Plastic deformation during the process is responsible for shaping the particles while fracture and cold-welding decrease and increase the particle size, respectively [35]. Photolithography is commonly known to be a rapid and cost-effective tool in developing nanoarchitectures with a limited resolution of 100 nm [36], [37]. In contrast, electron and ion-based lithography allow ordered nanostructured arrays with a resolution of up to 50 nm features with good control over particle shape and spacing.

On the other hand, laser ablations can generate various NPs such as semiconductor quantum dots, carbon nanotubes and core–shell NPs from organic solvents and water without stabilising agents. Metal submerged in a liquid solution can be irradiated by the laser beam, where the plasma plume is being condensed – nucleation and growth of laser-vaporised species produce NPs [38]. The extreme quenching of vapour produced high purity NPs of less than 10 nm is a reliable alternative in synthesising metal-based NPs [39]. As a type of surface treatment, chemical etching where nanoscale features/roughness can be created on the material’s surface by soaking it into an etchant such as hydrofluoric acid (HF) and sodium hydroxide (NaOH). The combination of an etchant and a surface-protecting agent such as polyvinyl pyrrolidone (PVP) or polyacrylic acid (PAA) has been utilised in producing hollow structured nanomaterials. The hollowing effect has been demonstrated as a general phenomenon for silica NPs [40]. Overall, top-down approaches can produce large quantities due to their comparable simple and straightforward destruction from bulk materials. However, the nanomaterials produced by top-down approaches are mainly polydispersed since size control is difficult.

In contrast, the bottom-up approach provides better control in particle morphology and size due to its building up from atom to clusters and nanomaterials. The bottom-up approach commonly includes sol–gel, spinning, chemical vapour deposition (CVD), biosynthesis, and pyrolysis. The sol–gel process involves the formation of an inorganic colloidal suspension (sol) and gelation of the sol in a continuous liquid phase (gel) to form a three-dimensional network structure [41]. This process involves three reactions: (1) hydrolysis, (2) alcohol condensation and (3) water condensation. Through this method, pure and homogeneous NPs such as metal, metal oxide, and ceramic NPs can be synthesised at low processing temperatures [42], [43], producing composites and complex nanostructures [44]. The synthesis of NPs by spinning requires a spinning disc reactor (SDR) with controlled temperature. The SDR is normally filled with nitrogen or other inert gas to avoid chemical reactions during spinning. The disc is rotated at different speeds causing the atoms or molecules to fuse and precipitate [45], determining the characteristics of synthesised NPs. CVD is the chemical reaction between the heated substrate and combined gas, depositing the gaseous reactant as a thin film on the substrate surface [46]. This method has great significance in generating carbon-based nanomaterials such as carbon nanotubes. The gaseous reactants decompose into carbon atoms and recombine into carbon nanotubes at high temperatures. The choice of catalyst significantly affects the morphology and type of nanomaterial obtained; for example, nickel (Ni) and cobalt (Co) catalysts provide multilayer graphene, whereas copper (Cu) catalyst provides monolayer graphene [47]. CVD produces highly pure, uniform, and strong nanomaterials and is well known to produce two-dimensional nanomaterials. However, the gaseous by-products are highly toxic [48].

Biosynthesis of biodegradable metallic nanomaterials with microorganisms such as bacteria, fungi, and algae is uncommon through mechanisms such as biosorption or bioreduction of aqueous solutions of metal salts via intra- or extracellular enzymatic activities [49]. Nonetheless, safety measures are essential when using pathogenic bacteria and fungi during the biosynthesis of NPs. For instance, photosynthetic bacteria such as Rhodopsuedomonas capsulata can be used to obtain gold NPs of 10–20 nm extracellularly while the bacterial enzyme Nicotinamide Adenine Dinucleotide Hydride (NADH) reductase plays a major role in reducing gold ions to gold NPs [50]. Favourably, the enzymatic activity of NADH-reductase causes long-term stability in the extracellular silver NPs produced by Fusarium oxysporum fungus [51]. Pyrolysis is commonly followed in industries for the large-scale production of NPs. The liquid/vapour precursor is fed into the furnace and combusted with either flame, laser or plasma under high pressure and high temperature. The NPs are recovered from the gaseous by-products. NPs synthesis by pyrolysis is simple, efficient, and cost-effective for high yield [35].

Cost-effectiveness is a major challenge in the synthesis of nanomaterials as well as the scaling up of production [36]. Many of the conventional methods are met with issues of low production rates. In contrast, the more economical ones, such as pyrolysis and mechanical milling, require intense operating conditions at extreme temperature and pressure or typically produce undesirable, highly defective NPs. The advantages and disadvantages of each synthetic method are summarised in Table 1. In comparison, US has been a useful tool for process intensification, particularly the cavitation effects eradicate the need for external energy resources and are more sustainable.

**Table 1:** A summary of various methods for the synthesis of nanomaterials.

Nanomaterials synthetis method	Mechanisms involved	Advantages and Disadvantages	Ref.
Mechanical milling	Impact of the ball-powder-ball collision on powder particles which undergo deformations and/or fractures, thus achieving size reduction and/or alloying	More economical for large scale processes; irregular particle shape and crystal defects are induced, creating a metastable composition	[34]
Lithography	Transfer of nanopatterns with templates for masked lithography and direct transfer for maskless approach. Another approach (nanosphere lithography) is through the dispersion of spherical colloids to form a colloidal crystal mask for selective patterning and allow deposition of desired materials	Can be top-down or bottom-up, generally high resolution of shape and position of NPs, yet production cost is high with low throughput	[36], [58], [59]
Laser-ablation	Removal of the surface atom from a solid target with a laser energy source. Nucleation of evaporated species and subsequent growth into NPs	A green method for controlled and high purity NP synthesis without using toxic chemicals, yet low production rates	[39]
Chemical etching	Chiseling of nanostructures out of solid surfaces through the use of etchants such as strong acids and/or alkalis	High etching rate and low equipment cost; difficulty in achieving smaller features and reliance on hazardous materials	[40], [60]
Sol-gel	Chemical method involving hydrolysis of precursors, polycondensation of molecules to form a network of colloids and subsequent aging, drying and calcination that controls the size and morphology of NPs	High control over NPs texture, achievable at a lower temperature; however, the processing time is long	[44]
Chemical vapour deposition	Vaccum deposition of vaporised precursors on heated substrates forming 2D NPs	High purity and homogeneity of the product. The process is limited by extremely high-temperature demand, and by-products are toxic gases	[46]
Biosynthesis	Use of microorganisms as bioagents, the enzymatic activities foster reduction and stabilization of NPs	Green and environmentally friendly approach, yet poor control over NPs size and many other mechanisms are still poorly understood	[49]
Pyrolysis	Thermal decomposition of precursors and subsequent formation and growth of particles by gas-phase chemical reactions and coagulation	High product yield and cost-effective but extreme synthesis conditions	[35]

The synthesis of NPs through the sonochemical route is similar to flash pyrolysis but with a much shorter duration (by > 10^4^) and higher thermal temperatures (5- to 10-fold) [52]. As US irradiation diffuses into the liquid medium, massive energy increases inside the bubbles (cavities), generating extremely high temperature and high pressure, leading to chemical excitation of the matter inside the bubbles and the surrounding of the bubbles during implosion. This method is useful in synthesising CoS_2_, alloys, oxides, and selenides like CdSe and ZnSe [53], [54], [55], [56]. Sonochemistry provides alternative fabrication for low-cost and high yields in a short reaction time, capable for large-scale production of high homogeneity nanomaterials [57], even at ambient conditions.

### 2.2 US-driven synthesis of nanobiomaterials

Ultrasonication is recognised as one of the earliest techniques for synthesising nanostructured materials [61]. It is even deemed superior to other synthetic methods, specifically in preparing amorphous products, nanomaterials into mesoporous materials, deposition of NPs on ceramic and polymeric surfaces, and controlling the morphology of nanomaterials [11]. The advantages of the US-assisted method include intensified reactions with high product yield in a short time and submicron effects of enhanced localised heat and mass transfer [62], [63]. The primary phenomenon induced by ultrasonication is bubble cavitation, as sonication energy induces the nucleation of impurities and gas in the liquid media into bubbles of tenths of microns in size which subsequently collapse [64]. The intense implosion then invokes distinct physical and chemical effects, where both have different impacts on the formation (and transformation) of nanomaterials.

The powerful US sets off various effects such as heat, agitation, diffusion, interface instabilities, friction, mechanical rupture, and chemical reactions. The collective mechanisms play multifaceted roles in the formation of nanomaterials. Notably, most literature reported synergistic US-assisted processes instead of exclusive US processes for material synthesis as sound energy enhances the efficiency and product yield than other methods. Different mechanisms and particle behaviours are induced by sonic waves depending on the reaction media and conditions [62]. The following sections are extensively categorised into US-induced physical- and/or chemical driven synthesis of NPs, and discuss the formation of nanobiomaterials via sonication, including lipid-based NPs, metal-based NPs and superparamagnetic NPs. Nanomaterials synthesis driven by the physical effects of US mainly results in surface modification, emulsification and exfoliation of layered materials such as graphites through cavitation induced high-speed jets and intense shock waves [65]. Chemical phenomena achieved through power US, or sonochemistry, generally trigger nanostructured materials in two regimes: gas-phase inside the collapsing bubbles or solution-phase outside the bubbles [10].

The application of power US for chemical processes intensification is fairly established, with earlier work on developing scale-up systems recorded since the mid-1980s [66]. Consequently, designs of various ultrasonic reactors and/or transducers may have arisen. However, as most work cited in this review are carried out at lab-scale with the use of generic ultrasonic processors with US horns or probes immersed in beakers and/or makeshift vessels, or even sonication bath to achieve batch sonication at small volumes, the engineering discussion on the design and system parameters of sonochemical synthesis of NPs in this review will be rather limited.

### 2.3 Simplistic view of sonochemistry theories and models

Despite the common use of US in experimental studies on nanomaterials synthesis, the augmentation of US for industrial application is still held back mainly by the challenge of controlling the sound field distribution and performance of the sonication process; as such, the resultant yield and morphology of products [67]. Additionally, sonochemical synthesis of NPs consists of complex phenomena of which some mechanisms may not have been fully understood, and literature on numerical simulations of the synthesis process, especially chemical reactions within and outside of bubbles are scarce, which otherwise would be useful when validating the physical and chemical effects of various sonication parameters. Nonetheless, theoretical models and numerical simulations of fundamental fluid dynamics inside a single collapsing bubble have partly elucidated the impact of sonication that seemingly agrees with the experimental observations and results and formed much of the basis of the subject [68]. One of the earliest attempts to investigate and quantify the phenomena in a cavitating bubble was through the study of light emission from a pulsating air bubble irradiated by US, single-bubble sonoluminescence (SBSL) [69] attributed to election-ion radiative recombination and electron-atom bremsstrahlung [70]. Likewise, most numerical studies have also adopted the SBSL model that assumes a spherical symmetrically oscillating bubble in water [71].

**Fig. 1:**
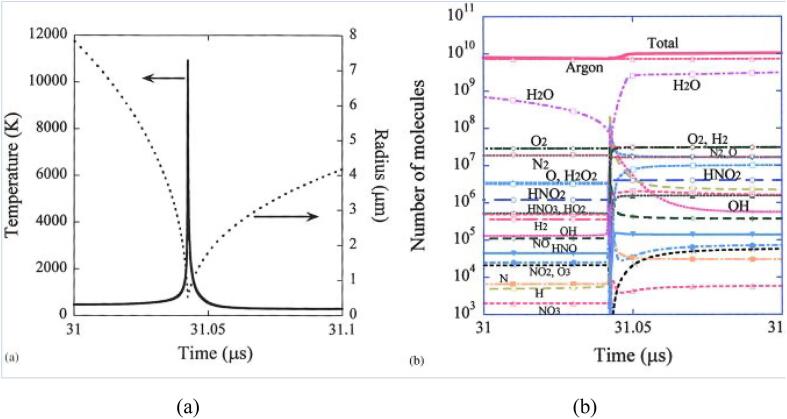
(a) Simulated changes in temperature and radius of an SBSL bubble at steady state, where the collapse occurred within 0.1 ms (b) Calculated results of the number of molecules inside a bubble based on the SBSL model. Various species are formed as high heat from the cavitation dissociates air and water vapour within the bubble. Reprinted from [72] with permission from AIP Publishing.

The expansion and collapse of a bubble are due to the rarefaction and compression of the US, and within the extremely brief moment of violent bubble collapse, a significant increase of temperature and pressure occurs, as calculated and indicated in Fig. 1a based on the SBSL model [72]. Early spectroscopic analyses of bubble luminescence by Suslick et al. revealed that localised hot spots of 5000 K and 1000 bar or even more extreme conditions are typically generated, achieving heating and cooling rates of more than 10^10^ K/s [10], [73]. According to Yasui [68], as opposed to suggestions that temperature and pressure are highest at the centre of a collapsing bubble, the temperature and pressure are nearly spatially uniform, with a variation of temperature at the bubble wall due to thermal conduction with the surrounding liquid. Studies indicate a peak in bubble temperature at lower ultrasonic frequencies (<100 kHz) corresponding to increasing acoustic amplitudes. Following bubble growth and increase in water vapour within the bubble after a violent collapse, temperature surges in the bubble, while the vapour fraction in bubble expansion at higher frequencies is less impacted [74].

Numerical simulations of nonequilibrium chemical reactions in SBSL also indicate that the main oxidant following the dissociation of water vapour and air inside the collapsing bubble is OH radicals, alongside other chemical species [72], [74]. Two other main oxidants have been identified as H_2_O_2_ and O atoms when the vapour fraction within the bubble is lower than 0.5. Calculations were performed to determine the dissolution and formation rate of OH radicals and other chemical species at the end of bubble collapse; the relative amount of each identified species is shown in Fig. 1b [72]. While chemists are certain that the cavitation effect and sound energy enhance most reactions, Jean-Louis Luche established certain guidelines to distinguish “true” and “false” sonochemistry, wherein reactions proceed via the radical or radical-ion pathways are considered as truly US-induced and suggested that other non-radical reactions should not be susceptible to sonication [75]. An extensive review was reported in 1989 that sought to unfold other characteristics of various reactions under the cavitation effect [76]. However, questions arise about sonication effects on chemical reactions as a whole, discounting bubble growth and implosive collapse that only affects 0.1% or less of the reaction medium [77]. Tuulmets et al. noted retardation in reactions as the US suppresses hydrophobic interaction of KCN-catalysed benzoin condensation of benzaldehyde in water and ethanol [78] and uncovered significant kinetic sonication effects in hydrolysis reaction in acetonitrile–water binary mixtures with reduced US power that presumably did not induce cavitation [77]. This is attributed to moderate energy from liquid microstreaming that purportedly shift solvation equilibria and stimulate subtle interactions in solutions, verifying kinetic ultrasonic effects in the absence of acoustic cavitation.

Numerical studies on a single bubble system are less complicated, especially when studying the chemical reactions offset by transient cavitation, than multi-bubble systems with more complex bubble behaviours. Comprehensive reviews have been reported on the theory and fundamental aspects of sonochemistry, including numerical analyses of sonochemistry [68], dynamics of bubble oscillation [70], and the nonlinear bubble dynamics of oscillation [79]. The mechanochemistry and reaction dynamics during cavitation [80], the influence of dissolved gas on the thermal conductivity, nucleation and stability of bubble [81] and the effect of parameters on the sonochemical process [82] were also investigated. US frequency and acoustic amplitudes are the primary factors directly impacting sonochemically active bubble sizes and consequently the amount of oxidants in the ambient bubbles of optimal sizes [83]. Frequency is indicative of the energy that sound waves carry. At the same time, acoustic amplitude defines the degree of change in sound wave compression and rarefaction and is proportional to the operating voltage. Typically a frequency of 20 to 40 kHz is sufficient to generate sonophysical effects such as microconvection and shockwaves. In comparison, high ultrasonic frequencies exceeding 150 kHz will induce chemical effects facilitating reactions with radicals [22]. Based on the numerical simulation of chemical reactions carried out by Merouani et al. at 200 to 1000 kHz to determine the effects of US frequency and acoustic amplitude on the size of active bubbles, the range of ambient radius for an active bubble was found to increase with increasing acoustic amplitude up to 3 atm [83].

Conversely, a decline in the bubble size was recorded with the increase of frequency. This result trend seemingly agrees well with other numerical and experimental studies of single and multibubble systems [74]. The following section focuses on the experimental work on the sonically prepared nanobiomaterials.

## 3 Nanobiomaterials synthesis induced by physical effects

Energy is dissipated in the form of viscous flow as acoustic waves pass through a medium [84], and the pressure variation generated in liquid media causes the cavitation of gas microbubbles. As the acoustic bubbles collapse, especially near solid surfaces, microjets and shock waves are released and propagate through the liquid medium [10]. The microjets then hit the solid surfaces at high speed, causing pitting and erosion [65], thus modifying and/or creating nanostructures. The aftermath of cavitation implosion generates turbulent flow and microstreaming as an effective means to mix liquids and facilitate interparticle collisions in the suspensions of solid particles in liquids [10]. With the special effects realised from the US, the obtainment of nanobiomaterials via US-assisted processes have been reported [11]. In the following discussion, the physical effects of US-driven synthesis of nanobiomaterials are based on materials exfoliation, biomolecules immobilisation on NPs, and NPs yielded from nebulisation.

### 3.1 US-induced exfoliation, dispersion and emulsification of nanobiomaterials

**Fig. 2:**
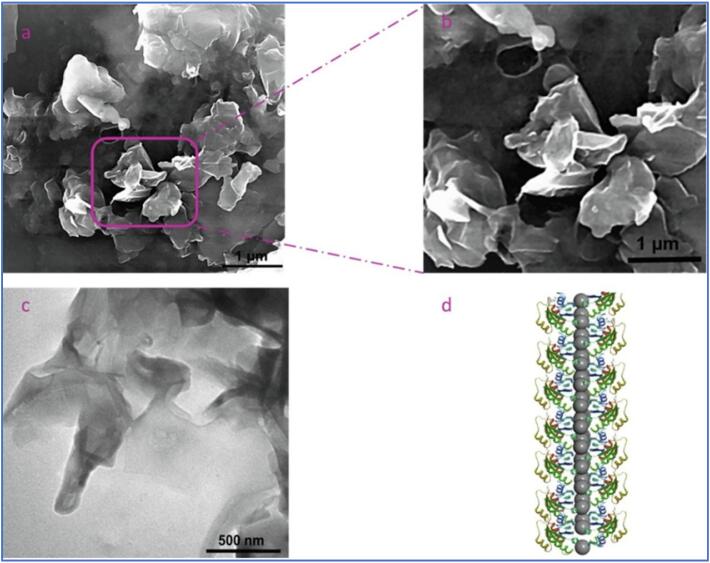
(a, b) SEM images of exfoliated graphene. (c) TEM image of Herceptin-graphene (d) Schematic image of the synthesised graphene. Reprinted from [39] with permission from the American Chemical Society.

Controlled ultrasonication coupled with liquid-phase exfoliation (LPE) is becoming a standard approach for the derivation of 2-dimensional (2D) nanomaterials like graphene and black phosphorus [85], [86], [87], [88], which have profound use in the biomedical and pharmaceutical fields such as wound healing and the development of nano-smart drug delivery systems for multi-mode cancer treatment [89], [90]. The exfoliation of stacked materials into mono- or few-layer nanosheets is driven by the mechanical action of inertial cavitation, involving short-lived cavitating bubbles undergoing vigorous and dynamic collapse that could radiate shockwaves with velocities up to 4000 m/s [85]. Tyurnina et al. investigated the key parameters of sonication for graphene flake in pure water. They identified the control over graphene quality, such as thickness and surface area, and production yield was subjected to US frequency, higher acoustic cavitation intensity, and uniform distribution of cavitation events in the sonicated volume [14]. At higher frequency and input power, the overall quality and yield of the few-layer graphene (FLG) improved with thinner flakes. At the same time, the defects were minimised with smaller size cavitation bubbles. Askari et al. produced Herceptin-stabilised graphene by exfoliating graphite flakes in the monoclonal antibody solution under sonication [91]. Herceptin is the most widely used targeted drug in the clinic, playing a profound role in breast cancer treatment [92]. The physicochemical analysis on the graphene indicated that the Herceptin residues were linked to graphene nanosheet surfaces through hydrophobic interactions and hydrogen bonding. The graphene production was improved with treatment time using constant US power (Fig. 2). The Herceptin-graphene was subjected to cytotoxicity evaluation with methyl thiazolyl tetrazolium (MTT) assay, illustrating no obvious toxicity against the 3-dimensional (3D) spheroid of skbr3 cells.

The dissipation of acoustic energy in liquid media is sufficient to generate interfacial turbulence to form a new interface and break macro droplets into fine emulsions exceeding 0.1 µm [93], [94]. Nirmala et al. reported on the potential use of cumin seed oil extracted from *Cuminum cyminum* for therapeutic uses [95]. Cumin oil nanoemulsions were prepared by first forming macromolecules of oil in water with Tween 80. The droplets were further broken down by high-energy shockwaves generated through sonication into nanoemulsions ranging from 10 to 150 nm based on the ratio of oil to surfactant and water and the sonication time. The cell viability test of cancerous tongue carcinoma (SAS) cell line and the non-cancerous (Hek 293) cell with cumin oil nanoemulsions via MTT assay have shown that cumin is a potential component for cancer therapy. Similarly, the nanoemulsions also exhibited significant antibacterial activity against *S. aureus*.

### 3.2 Preparation of superparamagnetic NPs and other metallic NPs through co-precipitation

**Fig. 3:**
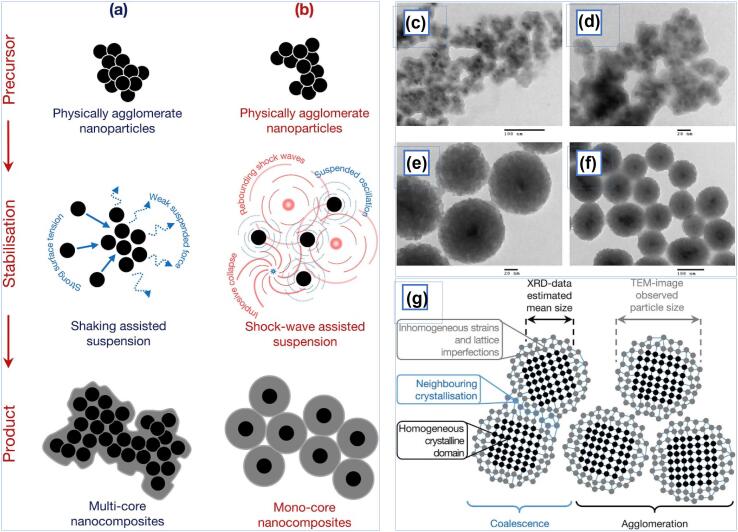
Formation illustration of Fe_3_O_4_ nanocomposites via (a) shaking assisted process and (b) US irradiation. TEM images of (c,d) multi-core nanocomposites show the composite having a polycrystalline core while (e,f) nanocomposites produced from US assistance exhibit clear mono core–shell structure, attributed to the diffusive and surface-protective shockwaves from implosive bubble collapse that form discrete core Fe_3_O_4_ NPs within silica. (g) In the US-assisted preparation of MNPs, it has been hypothesised that the shock waves generated from the collapse of the acoustic bubble counteract the high tension of MNPs, thus preventing crystalline coalescence on neighbouring surfaces that would yield heterogeneous-sized products. Reprinted from [97] with permission from Elsevier (CC-BY license).

Due to their biocompatibility and superparamagnetic properties, magnetite NPs (MNPs) have been widely employed in biomedical applications such as targeted drug delivery and magnetic resonance imaging (MRI) [96]. Bui et al. reported the synthesis of magnetic-silica nanocomposites via a two-step process of first synthesising Fe_3_O_4_ NPs followed by the Stober approach of hydrolysis of tetraethoxysilane (TEOS) in a solution of ethanol, ammonia, sodium silicate and the as-synthesised Fe_3_O_4_ powder [97]. The synthesis of Fe_3_O_4_/SiO_2_ NPs has always been plagued by the tendency of the particles to aggregate, thus forming large, heterogeneous composites with lower individual magnetite MNP. Two approaches were attempted to obtain Fe_3_O_4_/SiO_2_ nanocomposites by mechanically stirring the solution or subjecting it to ultrasonic irradiation. X-ray diffraction (XRD) and TEM analysis indicated that the co-precipitation synthesis of superparamagnetic NPs at a considerably low frequency of 42 kHz and 100 W, producing homogeneously spherical NPs. Also, the nanoscopic US effect of shock waves induced from the implosive collapse of acoustic bubbles provided a surface-protective effect that stabilised both magnetite MNPs and nanocomposites from spontaneous aggregation into a polycrystalline nanocluster, as shown in Fig. 3.

In contrast, the nanocomposites produced from mechanical stirring/shaking agglomerated due to weaker diffusive force. The as-synthesised nanocomposites were tested for superparamagnetic properties. The agglomerated samples exhibited a higher magnetisation of 50.2 emu/g due to the embedded magnetite core instead of quasi-mono US-derived nanocomposites, which displayed a magnetisation of 3.2 emu/g. Using the sonochemical approach, 3-amino propyl triethoxyl silane (APTES) was also successfully grafted onto superparamagnetic iron oxide NPs (SPION) at a minimum sonication period of 1 min [98]. The functionalisation of MNPs with amino and other functional groups improved the overall physicochemical properties of NPs and allows superior use for other biomedical applications [99]. According to Sodipo and Abdul Aziz, shockwaves generated from ultrasonic irradiation stimulated rapid silanisation reaction between the organo-silane molecule and the SPION, in addition to homogeneous sonochemistry within the localised hot spot [98].

Hydroxyapatite (HAp) is a bioactive polycrystalline bio-ceramic that can be used for bone tissue ingrowth [100], and recent studies indicated the composites of HAp and metallic oxides such as titanium dioxide (TiO_2_) improved the mechanical properties, providing additional support to the human body, and even stimulate cell growth [101]. The derivation of HAp/TiO_2_ nanocomposites via sol–gel and co-precipitation methods with ultrasonication at 20 kHz and 750 W yielded smaller NPs ranging from 17 to 20 nm with better crystallinity with an increase in the US irradiation time. Nikolaev et al. further expounded on the effect of US on HAp synthesis by studying parameters such as frequencies and intensities on the control of particle size [102]. They noted that frequencies have a minor effect on particle size. In contrast, vortices generated at non-cavitation regimes at the reaction sites have a more substantial effect on the crystal sizes than high-intensity cavitation regimes.

### 3.3 Nebulisation – Ultrasonic spray pyrolysis

Ultrasonic spray pyrolysis (USP) is another bottom-up approach for synthesising metal NPs using the US as the method of atomisation [103]. In principle, when the intensity of periodic vibrations set off by a piezoelectric crystal in the ultrasonic nebuliser is sufficiently high, droplets are propelled off the oscillating surface of the liquid, thus achieving the atomising effect [104]. Ultrasonically nebulised precursors, usually Newtonian fluids with low viscosities, are transported via carrier gas through a heated region [10]. The droplets undergo subsequent stages of evaporation, drying, chemical decomposition and sintering into fine powders or even hollow shells. Majeric and Rudolf produced a rather comprehensive review on the advances in USP processing of metal NPs. They elucidated in detail two USP mechanisms for forming NPs depending on the state of precursor: Droplet-To-Particle (DTP) and Gas-To-Particle (GTP). In contrast to other methods of NPs production, USP is considered a far more cost-effective approach and can be easily scaled up to the industrial level with acceptable productivity in droplet sizes [105].

**Fig. 4:**
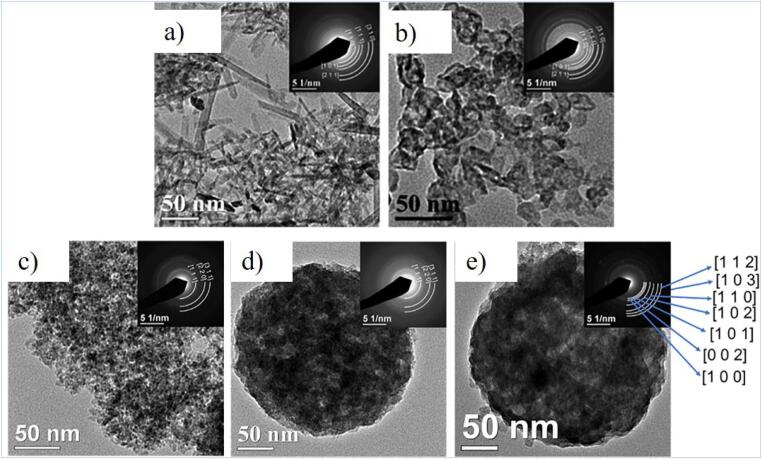
TEM images of (a) rod-like and (b) spherical ZnF NPs (c) pristine ZnS (d) sphalerite-rich N-doped ZnS and (e) wurtzite-rich N-doped ZnS NPs. Insets are Selected Area Electron Diffraction (SAED) patterns corresponding to XRD patterns. Reprinted from [106] with permission from Elsevier.

Malekzadeh et al. successfully employed a laser pyrolysis reactor with the customised ultrasonic atomiser and obtained zinc (Zn)-containing NPs having applications in nanomedicine [106] and wound care [107]. Zn acetate dihydrate solution was used as the Zn precursor. In contrast, thiourea, thioacetamide and sulphur hexafluoride (SF_6_) were used as sulphur precursors and fluorine sources respectively to form spherical and rod-like pristine and doped Zn sulphide (ZnS), Zn oxide (ZnO), and Zn fluoride (ZnF) NPs and the TEM images of Zn NPs are shown in Fig. 4. The ultrasonically atomised precursor mixture decomposition was achieved via focusing continuous wave CO_2_ laser beam at 65–85 W. The operating parameters (carrier and sheath gas flow rates) varied for different Zn NPs. SF_6_ was also used as a photosensitiser to assist the precursor solution to absorb the laser energy. The morphology and elemental distributions of the NPs were also studied. They concluded that the GTP mechanism led to the synthesis of pristine ZnO, ZnF and ZnS NPs, while nitrogen-doped ZnS used thiourea as both sulphur and nitrogen sources were synthesised via DTP.

Aizawa et al. and Nakagawa et al. adopted USP to synthesise apatite materials in the range of 0.5 to 3.0 μm as anti-angiogenic chemoembolisation agents and immunoceramics respectively for cancer therapy [108], [109]. Biodegradable hollow calcium phosphate microspheres (CPMs) were obtained by spraying the salt solution using an ultrasonic vibrator at 2.4 MHz into two furnace segments of 300 °C and 850 °C for drying and pyrolysis [108]. The freeze-dried CPMs were then loaded with an anti-angiogenic agent (TNP-470) that inhibited tumour vasculature formation. Biological evaluations were carried out to investigate the anti-angiogenic activity *in vivo* and *in vitro* of the microspheres using human uterine sarcoma FU-MMT-3 cells. Overall, TNP-470 loaded CPMs with smaller particle sizes demonstrated more significant tumour growth inhibition. In contrast, mixed-sized CPMs achieved better *in vivo* results, attributed to the broader embolisation of arteries and tumour microvessels of varying sizes.

Immunoceramics made of boron-containing apatite were studied as an inexpensive alternative to support cancer immunotherapy and possibly activate lymphocytes around cancer cells [109]. Boronic acid-containing polymers were found to have similar activity as plant-derived protein lectin, effective for lymphocyte proliferation. USP has proven to be an operative technique to synthesise spherical HAp and boron-containing apatite (BAp) particles of 0.5–1.5 µm diameter with complex composition. The approach is similar to producing CPMs, except the furnace temperatures were set at 400 and 1000 °C. HAp and BAp ceramics were formed by compacting and firing the USP-derived powder at 1200 °C, and splenocytes from female mice were seeded and cultured on the bioceramics. Flow cytometric analysis indicated that BAp ceramic with a higher BO_2_ group had more helper T cells and killer T cells. The cell morphology was affected by the immunostimulation from BAp, causing cell adhesion and activated lymphocyte activity.

## 4 Ultrasonic-driven chemical reactions

During the implosions of the cavitating bubbles, extreme conditions of heightened pressure and temperature of up to 1000 bar and exceeding 5000 K instigate chemical reactions within and surrounding the bubbles, all within an extremely short span of reaction time [64]. While there is no direct molecular interaction between the US and the chemical species, chemical reactions are driven by the acoustic energy or hot spots of collapsing bubbles [10]. The localised heat generated is sufficiently high to break all chemical bonds, producing radical species that initiate various reactions such as cross-linking monomers. The crystallinity of the product depends on the types of precursors, as amorphous NPs are formed from volatile compounds with reactions predominant in the gas phase. In contrast, sonochemical reactions occur in the liquid phase for non-volatile precursors producing either nanoamorphous or nanocrystalline particles. The reaction mechanisms occurring inside and around the cavitational implosion are entirely different, with the former referred to as primary sonochemistry as volatile gases present inside the collapsing bubbles can undergo reduction and the latter as secondary chemistry with the diffusion and reaction of radical species with solutes in the surrounding medium [10], [11]. The amorphous structure is formed in the collapsing bubble limited by the short lifetime of the cavitation with rapid cooling hence constrained growth of nuclei.

Extensive studies have been carried out to investigate the parameters affecting the yield of NPs, such as the impact of acoustic frequency and pressure on the generation of radicals, the properties of liquid media and dissolved gases, and even the dynamics of bubbles [110]. While the physical effects of US irradiation, such as causing surface defects and emulsification, typically occur at lower frequencies, sonochemical effects are more dominant at intermediate frequencies of 200–500 kHz [64], [84]. A greater number of radicals are generated at higher frequencies, thus enhancing the rate of reaction. In the following section, nanobiomaterials formed through sonochemical reactions have been highlighted and are broadly categorised as lipid-based and water-soluble carriers.

### 4.1 Sonoproduction of lipid- and carbohydrate-based NPs

Liposomes and micelles are lipid-based NPs with liposomes ranging from unilamellar to multilamellar vesicles [111]. At the same time, polymeric micro- and nanospheres are particles composed of natural biopolymers (proteins, polysaccharides) and other biocompatible synthetic polymers encapsulating core materials such as drugs or contrast agents [112]. Suslick and Grinstaff were among the earliest to report using the high-intensity US to produce spherical proteinaceous microcapsules containing non-aqueous liquid [113]. They attributed the cross-linking of bovine serum albumin (BSA) microcapsules to the disulphide bonds formed between protein cysteine residues initiated by sonochemically-induced superoxide. However, the sonoproduction of oil- and gas-filled protein microspheres relies on both the physical and chemical effects of US [10]. The two main phenomena induced by sonication are the emulsification of polymeric material, followed by acoustic cavitation, generating radicals that behave as cross-linking agents [112]. As mentioned earlier, the size distribution of microspheres produced is largely dependent on sonication parameters such as frequency, intensity and time.

**Fig. 5:**
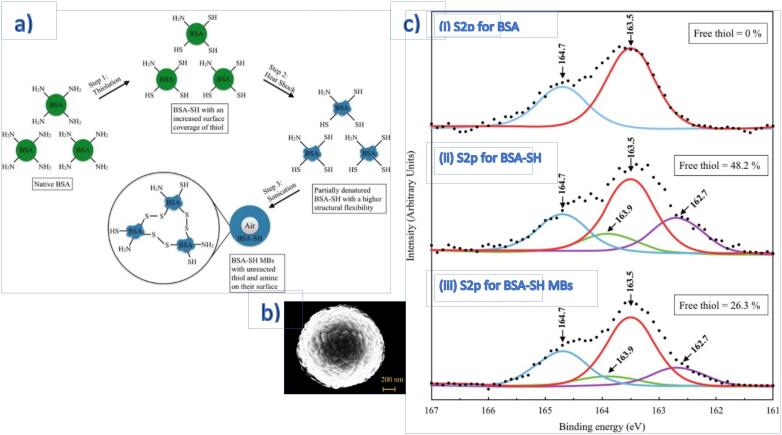
(a) Schematic and the flow chart exhibiting the synthesis of surface-treated BSA MBs (BSA-SH-MBs) (b) SEM image of spherical BSA-SH-MBs (c) High-resolution XPS spectra of S2P for (ci) BSA protein before surface treatment (cii) BSA-SH post-treatment and (ciii) cross-linked MBs BSA-SH-MBs illustrate an increase in free thiol after treatment and decreased after cross-linking of the microsphere. Reprinted from [114] with permission from Elsevier.

One of the limitations for wider use of conventional gas-filled microbubbles (MBs) is the short lifetime of the bubbles, which typically lasts only a few hours without stabilisers. With Traut’s reagent, long-lived air-filled MBs made of surface-treated protein were sonochemically produced at 20 kHz [114]. Traut’s reagent was added in molar excess of BSA, which was proportional to the degree of thiolation of BSA to render free thiols onto the surface of BSA. Superoxide generated from acoustic cavitation led to cross-linking of free thiols of cysteine residues to form disulphide bonds. A higher disulphide content contributed to thicker shells and longer-living MBs. X-ray photoelectron spectroscopy (XPS) spectra of S2p in Fig. 5 shows that following the surface treatment with Traut’s reagent, the amount of free thiol increased to 48.2% but reduced to 26.3% after cross-linking. The shell thickening with bubble shrinkage was also observed, attributed to the balancing of Laplace pressure, and the MBs remained stable for at least six months.

Skoll et al. attempted encapsulating biocompatible plant oil in human serum albumin (HSA) and the embedding of wheat germ agglutinin (WGA) to the capsule shell, which exhibits a high affinity for urothelial cancer cells [115], promising as a targeting molecule for cancer drug delivery. Proteinaceous nanocapsules with different plant oil core (olive, linseed, cotton seed, almond and rapeseed oil) size ranging from 662 to 862 nm with a polydispersity index (PDI) of 0.11 to 0.24 were sonochemically produced through the generation of perhydroxyl radicals that oxidize cysteine residues of the protein chains. Particles with WGA/HSA are slightly smaller in size, with fewer amino groups of HSA detected with the embedding of the fluorescently labelled targeting molecules.

Starch-based NPs (SNPs) have garnered significant attention in recent years due to their good biocompatibility that renders them good biocarriers for drugs and even food bioactives [116]. The effects of sonochemical production of SNPs from cornstarch are currently being investigated, with waxy starch or starch granules first disintegrated in solvents under sonication, followed by the conversion of amylopectin branches into amylose blocks under the influence of acoustic cavitation [117], in contrast to conventional starch acid hydrolysis. US treatment of waxy starch generally resulted in amorphous SNPs of 30–60 nm; however, Garcia-Garcia-Gurrola et al. obtained spherical or oval-shaped NPs [117] instead of platelet-like NPs as described by Boufi et al. [118]. The succinylated particles form micelles for octenyl succinic anhydride (OSA)-modified NPs were obtained from single-step US treatment. The ultimate size corresponds to the degree of substitution (DS), with larger NPs and higher surface charge obtained at higher DS. Hasanvand et al. indicated that the size of SNPs is largely dependent on the amylose to amylopectin ratio in the type of starch used, among other factors [119]. At the same time, it is known that a longer sonication time results in smaller particle sizes due to the force-induced scission of covalent bonds.

### 4.2 Sonochemical synthesis of microgels

**Fig. 6:**
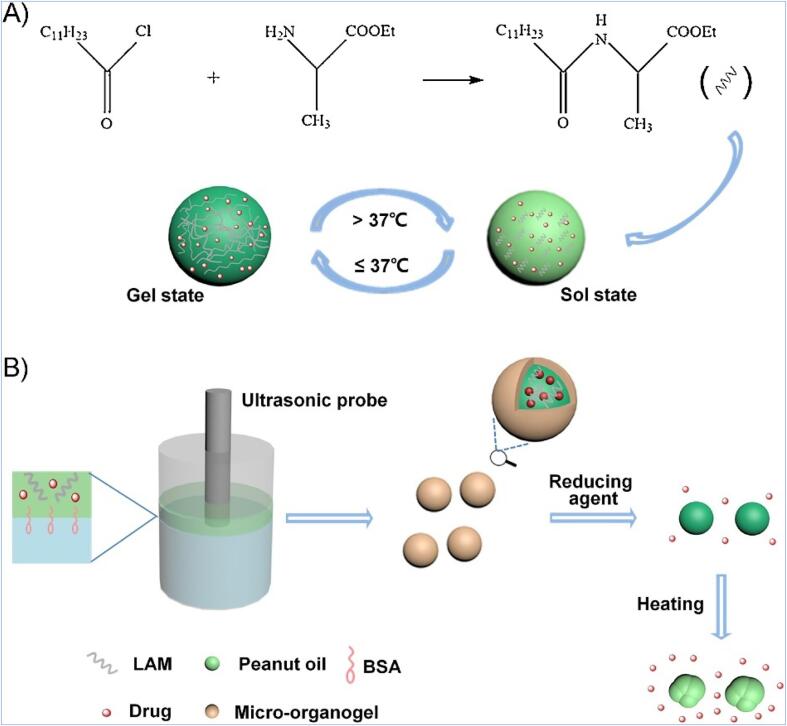
Schematic illustration of (A) sol–gel of gelator LAM through the reaction between acyl chloride and esterified L-amino acid, where the aggregates formed upon cooling (B) sonochemical assembly of drug- encapsulated BSA-MBs and stimuli-triggered release of the drug-loaded micro-organogel. Reprinted from [120] with permission from Elsevier.

Nanogels are systemic drug carriers with three-dimensional network structures that can carry hydrophobic or hydrophilic drugs and even biomolecules such as nucleic acids [120], [121]. Nanogels are considered hydrogels if the cross-linked polymer chains are water-soluble or swellable [122]. A water-loving micro-organogel with thermo- and redox-sensitive properties encapsulating hydrophobic drug was successfully synthesised sonochemically (Fig. 6). The organogel was formed from the self-assembly of n-lauroyl l-alanine methyl ester (LAM) gelator in the organic phase of medical peanut oil through hydrogen bonding. Notably, the LAM aggregates were thermo-reversible. Fluorescent dyes SIII or 7HC were used as the models of hydrophobic drugs and were encapsulated with BSA molecules acting as an amphiphilic stabiliser in the biphasic solution. However, strong oxidants generated during ultrasonication initiated sulfhydryl-crosslinking of the BSA shell. The size of the micro-organogel depends on the concentration of BSA, sonication energy and oil-to-water volume ratio; by optimising all these factors, the mean size of the product was less than 600 nm.

Shekar et al. described the encapsulation of Simvastatin, an antihyperlipidemic drug used for the prophylactic treatment of obesity in mucoadhesive microspheres for gastroretentive drug delivery [123], [124]. US-assisted microencapsulation via ionic gelation was performed by adding a solution of Simvastatin and calcium chloride to sodium alginate solution followed by chitosan solution. The synthesis was carried out under continuous sonication with a probe sonicator at 20 kHz and 130 W, while a similar procedure was repeated without sonication for comparison. Chitosan cross-linked alginate had good mucoadhesive properties [125]. It is suitable for entrapping Simvastatin, with ultrasonication improving drug dispersion and inducing cavities around the polymer structure, thus attaining high drug encapsulation. The results indicated that mucoadhesiveness of the microspheres increased with the concentration of chitosan, and higher yield and smooth and smaller sized particles were produced with increased sonication time.

### 4.3 Sonochemical reduction of silver, gold and other metal NPs

Nanostructured metals are produced through chemical reduction of non-volatile precursors enhanced by US irradiation which typically produces highly reactive radicals [65]. Fatimah et al. used *Clitoria ternatea* flower (CTF) extracts as a reducing agent for the biosynthesis of silver and gold NPs (AuNPs), which are the promising antibacterial and drug delivery agents [126]. *Clitoria ternatea* (L.) has high flavonol compounds and is thus reported to have antioxidant activity. A comparison was made against the effectiveness of reduction of auric chloride (AuCl_3_) and silver nitrate (AgNO_3_) in CTF extract into AuNPs and silver NPs (AgNPs) respectively via two methods: reflux for 2 h versus US irradiation for 30 min, both methods accelerated the collision of Ag^+^ and Au^3+^ with bioreduction in the CTF extract. Generally, both NPs generated under sonication were smaller and more homogeneous, 5–20 nm for AgNPs and 20–60 nm for AuNPs. Sonication at 40 kHz and 68 W induced the formation of free radicals, which initiated reduction and the proposed mechanism is as follows (Eqs. (1) to (8)):

(1) $$nH_{2}O+\left(US\right)\to \left(HÂ\cdot +Â\cdot OH\right)$$

(2) $$\mathrm{Ag}{\mathrm{NO}}_{3}\to {\mathrm{Ag}}^{+}+{{\mathrm{NO}}_{3}}^{-}$$

(3) $$\mathrm{OH}+RH\to RÂ\cdot +H_{2}O$$

(4) $$RÂ\cdot +{\mathrm{Ag}}^{+}\to Ag{}^{\circ}+R^{\text{'}}+H^{+}$$

(5) $$H{}^{\circ}+{\mathrm{Ag}}^{+}\to Ag{}^{\circ}+H^{+}$$

(6) $${\mathrm{Ag}}^{+}+H_{2}O\to Ag{}^{\circ}+Â\cdot OH+H^{+}$$

(7) nAg°→Ag°(aggregates)

(8) $${{\mathrm{Ag}}^{+}}_{n}\to {\mathrm{Ag}}^{-1}+{\mathrm{Ag}}^{+}$$

XPS analyses on both NPs indicated the presence of a capping agent, which was identified to be one of the active compounds of CTF. The calculated crystal sizes of the US formed AgNPs and AuNPs were 19.98 and 24.09 nm, respectively, slightly smaller than those formed via reflux. The antibacterial tests were carried out against *Escherichia coli*, *Staphylococcus aureus*, Klebsiella pneumoniae and Streptococcus pyogenes. Both NPs exhibited good antibacterial properties against *E. coli* and *S. aureus*, and the antibacterial activity of AgNPs against *S. aureus* was higher. At the same time, the activity of AuNPs was higher for *E. coli*. Using a similar approach, Jackson et al. recorded the use of two different Kenyan plant extracts, namely *Bridelia micrantha* and *Adansonia digitata* leaves, for the sonochemical synthesis of AgNPs with antibacterial effect; both showed good antibacterial properties against *E. coli* [127], [128].

**Fig. 7:**
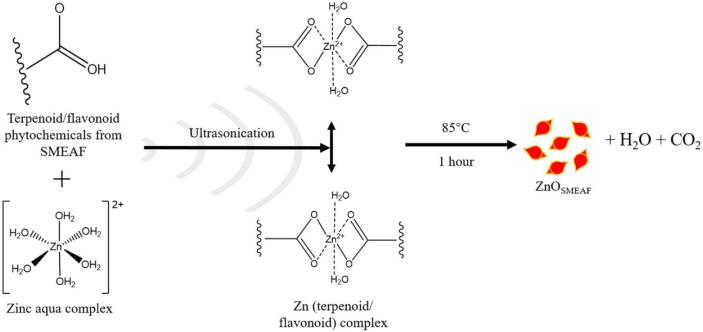
Proposed mechanism for the US-assisted bioreduction of zinc salt precursor into ZnO nanorice with phytochemicals from SMEAF. Reprinted from [129] with permission from de Gruyter (CC-BY license).

The biosynthesis of ZnO nanorice with anticancer activity has been described by Low et al., who used *Swietenia macrophylla* (*S. macrophylla*, or SMEAF), a medicinal plant reportedly having anticancer, anti-inflammatory and antitumour properties [129]. SMEAF seed extract was added to the Zn precursors, and the mixture was subjected to ultrasonication repeatedly, followed by post-treatment (Fig. 7). Two primary reactions took place; a double exchange of zinc nitrate precursors with NaOH to form zinc hydroxide followed by the decomposition of zinc hydroxide to ZnO, as shown below.

(9) $$\mathrm{Zn}{\left({\mathrm{NO}}_{3}\right)}_{2}∙6H_{2}O+2NaOH\overset{\Delta }{\to }Zn{\left(OH\right)}_{2}+2Na{\mathrm{NO}}_{3}$$

(10) $$\mathrm{Zn}{\left(OH\right)}_{2}+Na{\mathrm{NO}}_{3}\overset{SMEAF,\Delta }{\to }{\mathrm{ZnO}}_{\mathrm{SMEAF}}$$

**Fig. 8:**
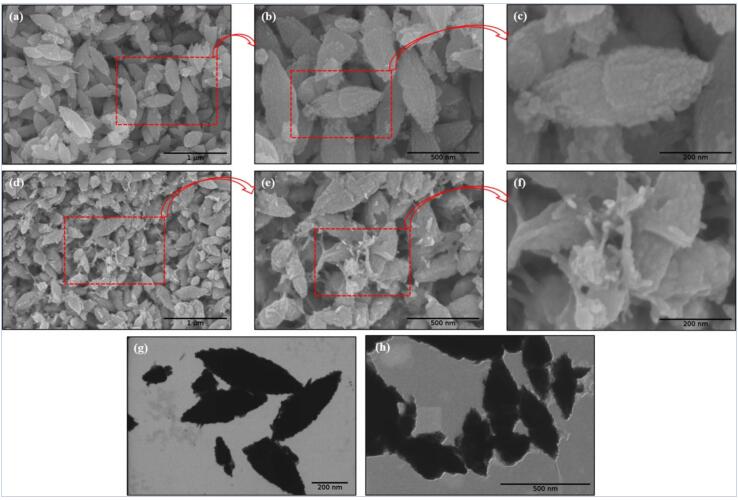
Biological reduction of Zn ions under sonication yielded ZnO with nanorice structure. FESEM micrographs of (a-c) ZnO_Chem_ (d-f) ZnO_SMEAF_ retained a similar structure with the addition of SMEAF; STEM micrographs of (g) ZnO_Chem_ (h) ZnO_SMEAF_ of scale bar 200 and 500 nm, respectively. Reprinted from [129] with permission from de Gruyter (CC-BY license).

**Fig. 9:**
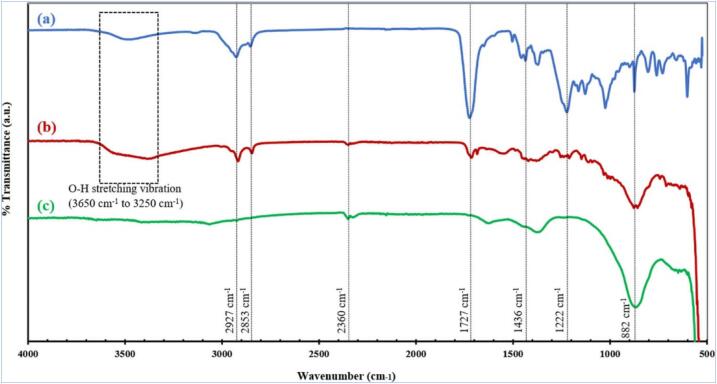
FTIR spectra of (a) SMEAF (b) ZnO_SMEAF_ and (c) ZnO_Chem_. Phytochemicals in SMEAF, particularly OH– groups in flavonoids, behave as reducing and capping agents controlling the size and morphology of ZnO NPs as indicated in the wide peak at 365 and 3250 cm^−1^ from O-H stretching vibration. Reprinted from [129] with permission from de Gruyter (CC-BY license).

US-enhanced reduction of Zn ions with flavonoids in SMEAF as the reducing and capping agents has proven to generate uniform ZnO NPs shaped like short nanorice (Fig. 8), with sizes ranging from 262 to 311 nm with the PDI of 0.166 to 0.402. ZnO NPs with SMEAF (ZnO_SMEAF_) as opposed to ZnO without SMEAF (ZnO_pure_), are larger due to the addition of the bioextract, which was verified through FTIR analyses indicating the peaks of phytochemicals such as alkyl compounds (2927 cm^−1^), ester carbonyl (1727 cm^−1^) and aromatic compounds (1222 cm^−1^), as shown in Fig. 9. ZnO NPs possess inherent cytotoxicity to cancerous cells. The treatment of HCT-116 colon cancer cells with ZnO_SMEAF_ exhibited a significant decrease in cell viability which is highly dependent on the dosage of the bioextract.

### 4.4 Ultrasonic production of other nanocomposites

**Fig. 10:**
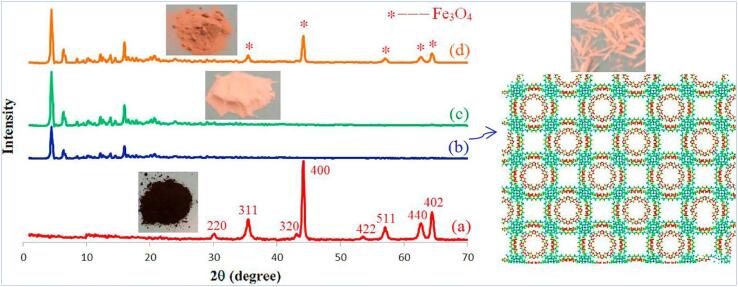
Powder X-ray diffraction (PXRD) analyses of (a) Fe_3_O_4_ NPs (b) bio-MOF single crystal (c) bio-MOF nanostructures, indicated higher crystallinity of pre-synthesised NP and sonochemically produced bio-MOFs (d) magnetic bio-MOF nanocomposites retained similar diffraction peaks as prior to the addition of Fe_3_O_4_, showing no obvious effect on the bio-MOF framework. Reprinted from [130] with permission from Elsevier.

**Fig. 11:**
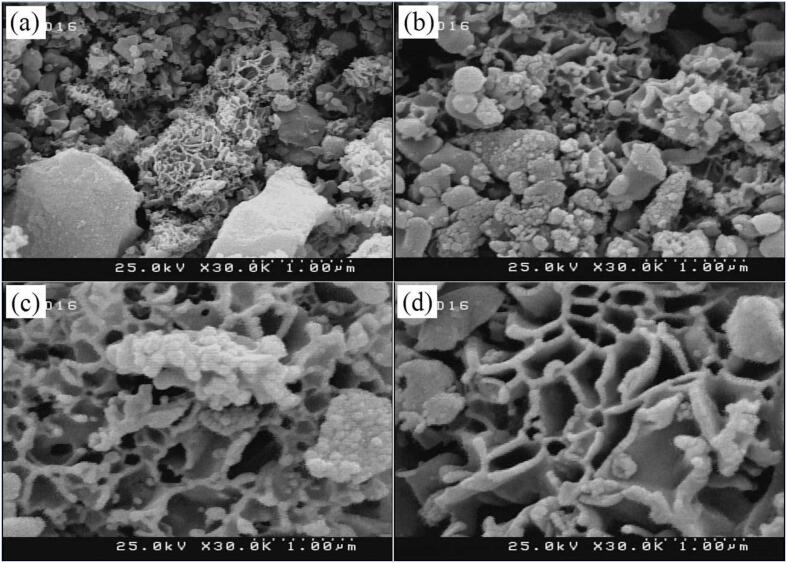
FESEM images of sonochemically synthesised Fe_3_O_4_@bio-MOF nanostructures at various times of sonication (a) 30 min (b) 60 min (c) 90 min and (d) 120 min. Sonication of up to 120 min was deemed the optimal time parameter that produced a non-agglomerated porous-layer open morphology. In contrast, different sizes and agglomerated Fe_3_O_4_ particles were observed within the shorter period. Reprinted from [130] with permission from Elsevier.

US-assisted synthesis of other nanobiocomposites have been reported, in particular a novel magnetic bio-metal–organic framework (MOF) nanocomposite effective against the parasitic activity of *Leishmania major* [130]. Nanostructured porous bio-MOF (Zn_8_(Ad)_4_(BPDC)_6_O.2(NH_2_(CH_3_)_2_)^+^, 8DMF, 11H_2_O) with Zn (II) ions, biomolecular adenine and 4,4′-biphenyl dicarboxylic acid (BPDC) as the building blocks and bridging ligands were prepared under sonication replacing the conventional solvothermal approach. Under acoustic cavitation, localised hotspots support the in situ metal/ligand reactions otherwise achieved via solvothermal route. A comparison of bio-MOFs obtained via both methods indicated an agreeable single-crystal XRD pattern with substantially shorter reaction time (2 h versus 24 h) and lower temperature (room temperature versus 130 °C) for the sonochemical process, as shown in Fig. 10. To obtain superparamagnetic Fe_3_O_4_@bio-MOF, the process was repeated by subjecting MAA-functionalised Fe_3_O_4_ with Zn, adenine and BPDC mixture to the same sonication conditions. A non-agglomerated porous-layer open morphology of magnetic bio-MOF as exhibited in Fig. 11d was obtained following prolonged sonication time resulting in higher morphological uniformity of Fe_3_O_4_ distribution on the surface of bio-MOF. The cytotoxicity effects of bio-MOFs were examined *in vitro* by determining the effects on promastigotes, intracellular amastigotes and J774 macrophages with MTT assays, and *in vivo* by treating BALB/c mice carrying Leishmania major. The results denoted suppression of *L. major* promastigotes and amastigotes in mice and reduced cutaneous leishmaniasis lesions on infected BALB/c mice.

Synthetic polymer-based nanocomposites, like bio-based polymers [131], have been widely used in biomedical applications such as the synthesis of bone cement [132] and in filling dental cavities [133]. Poly(methyl methacrylate) (PMMA) polymer nanocomposites with Fe_3_O_4_ as nanofiller were ultrasonically synthesised via a two-step process [134]. Fe_3_O_4_ NPs was first produced through co-precipitation of Fe(II)/Fe(III) salt mixture under the synergistic effect of US that formed H and OH radicals during transient cavitation·H_2_O_2_ generated through the recombination of OH radicals enhanced the oxidation of Fe^2+^ to Fe^3+^ ions. The Fe_3_O_4_ particles synthesised under the sonication effect were smaller (75.1 nm) in size than NPs generated through conventional mechanical stirring (286.1 nm). Fe_3_O_4_ NPs were treated with sodium dodecyl sulphate (SDS) surfactant for stability against agglomeration prior to emulsion polymerisation with methyl methacrylate (MMA) monomer. The nanocomposites had a mean particle size of 72.9–119.6 nm with good homogeneity. The measurements of the magnetic parameters of the PMMA/Fe_3_O_4_ nanocomposites showed a sharp reduction in the saturation magnetisation after the magnetic Fe_3_O_4_ NPs were dispersed in the polymer matrix, while the coercivity of Fe_3_O_4_ NPs increased. In conclusion, the nanocomposites exhibited enhanced thermal, mechanical and magnetic properties with a low loading of ≤ 5 wt% ultrasonically produced Fe_3_O_4_ NPs [128]. Table 2 summarises the US-driven synthesis of nanobiomaterials based on different physical and chemical mechanisms due to sonication.

**Table 2:** A summary of US-driven synthesis of nanobiomaterials based on varying sonication-triggered physical and chemical mechanisms.

Nanobiomaterials and their potential biomedical application	US-assisted synthesis	Sonication conditions	Product yield	Ref.
Herceptin-stabilised graphene for tumour-targeted drug delivery	US-assisted one-step graphite exfoliation and stabilisation of Herceptin	Sonication with an ultrasonic probe at 15 W with solution placed in an ice bath, and sonication time ranged from 30 min to 30 h.	Less than four layers of the graphene sheet	[91]
Cumin oil nanoemulsions for cancer therapy	US-assisted emulsification	Ultrasonicator with 750 W input power, 20 kHz for 5, 10 and 15 min	Diameter ranges from 10.4 ± 0.5 nm to 149.33 ± 1.15 nm	[95]
Mono-core magnetite-silica nanocomposites for targeted drug delivery and MRI	Co-precipitation with surface-protection effect preventing NPs agglomeration	Cleaner-type ultrasonication bath 42 kHz, 100 W	Average diameter 100 nm	[97]
Amino-silane functionalised SPION	Sonochemical functionalising of APTES on SPION under high-speed collision induced by shock waves	20 kHz Vibra-Cell, 750 W ultrasonic horn in an ice bath at 1, 10 and 20 min of sonication time	Diameter ranges from 2 to 25 nm	[98]
HAp/TiO_2_ nanocomposite for bone tissue ingrowth	US-assisted sol–gel and co-precipitation	Ultrasonic processor at 20 kHz and 750 W with one pulsation per 2 s, at time intervals of 15, 30, 45 and 60 min	Semi-spherical and agglomerated, 17–20 nm	[101]
Zn-containing NPs for nanomedicine and wound care	USP	Zn precursor atomised by ultrasonic atomizer (metal mesh) into the reaction zone of laser pyrolysis where the precursor mixtures were decomposed under continuous-wave CO_2_ laser beam at a wavelength of 10.6 μm and laser power of up to ∼ 100 W, the temperature of reaction zone was estimated to range from 800 to 1400 °C	Spherical and rod-shaped particles	[106]
Calcium phosphate microspheres as anti-angiogenic chemoembolisation agent	USP	Solution was sprayed into a two part-furnace (300 and 850 °C) with an ultrasonic vibrator at 2.4 MHz	0.5–3 μm	[108]
HAp and BAp immunoceramics	USP	Ultrasonic vibrator at 2.4 MHz generated droplets that were transferred by air flow into the heating zone of the furnace having two different temperatures (400 and 1000 °C)	Hollow spheres of 0.5–1.5 μm	[109]
Reactive MBs of surface-thiolated BSA	US-induced formation of disulphide bonding on BSA	Sonic dismembrator at 20 kHz was inserted into a jacketed beaker. US impulse of 23% amplitude applied for 45 s	Spherical MBs of 2.5 μm and shell thickness 155 nm	[114]
Proteinaceous HSA nanocapsules with biocompatible plant oil cores	US-induced generation of perhydroxyl radical oxidises cysteine residue	Micro-tip of Bandelin HD 2070 sonifier placed at the interface of the two-phase mixture, sonication with an acoustic power of ∼ 253 W.cm^−2^ at 40% amplitude for 2 min	Capsule sizes ranging from 655 to 862 nm, PDI 0.11 to 0.3	[115]
Water-loving drug-loaded micro-organogel with thermo and redox-sensitive behaviour	Sonication initiated sulfhydryl-crosslinking of BSA shell	Sonication at 20 kHz in pulse sonication mode, insertion of an ultrasonic probe into layered biphasic solution contained in a cylindrical tube. The temperature was maintained at 37 °C as organogel was thermo-sensitive	Spherical, average diameter of 800 nm, range of 300 nm to 2.0 μm	[120]
Simvastatin encapsulated in the sodium alginate and chitosan microsphere for gastric retention and controlled delivery	US-induced cross-linking of polymer coating	Continuous sonication at 20 ± 3 kHz, ultrasonic power of 130 W and temperature control at 50 °C with 2 cm probe sonicator immersed in a glass beaker	0.92 to 3 μm	[124]
Starch NPs as biocarriers	Disintegration, esterification and emulsification of starch under the US	Ultrasonic processor of 500 W nominal power at 20 kHz with titanium probe coupled with a water bath to maintain the temperature at 25 °C. Amplitude of 85% with a different exposure time of 20 min interval	48 to 83 nm, PDI of 0.224 to 0.91	[117]
AgNPs and AuNPs as promising antibacterial agents and for drug delivery	Sonochemical reduction	30 min US irradiation with US probe at 40 kHz fand 68 W	AuNPs: 10–50 nm, AgNPs: 5–20 nm	[126]
AgNPs with antibacterial effect against *E. coli* and *S. aureus*	Sonochemical reduction	Sonicator bath of 20 kHz until no further color change	Average diameter 16.07 ± 3.192 nm, 13 nm	[127], [128]
ZnO nanorice with anticancer activity	Sonochemical reduction and immobilisation of SMEAF	5 cycles sonication of 15 s each with a 12 s break with US horn in a water bath at 45 W	Mean size of 311 nm, PDI of 0.402	[129]
Fe_3_O_4_@bio-MOF effective against leishmaniasis	Combined US effects for linking of bio-MOF building blocks and distribution of Fe_3_O_4_ NPs	Mixture was sonicated in an ultrasonic bath at ambient temperature for 30 min at 40 kHz and using 30 and 60 W	Surface area of 1096 m^2^.g^−1^, pore size 13–14 Å	[130]
PMMA/Fe_3_O_4_ nanocomposites	Two-step synthesis: co-precipitation of salt mixtures and in situ polymerisation through free radicals formed during transient cavitation	Continuous sonication of the reaction mixture for 30 min under nitrogen atmosphere at 20 kHz using a probe-type micro-processor	Average diameter of 76 to 116.6 nm	[134]
HAp nanoflowers for protein adsorption	Self-assembly of HA nanosheets	Continuous US irradiation at 28 kHz with 200 W for 40–90 min	Surface area of 77.3 to 181 m^2^ g^−1^	[135]
Chitosan microspheres and other responsive block copolymers microspheres	Crosslinking of chitosan via sonochemically generated radicals in aqueous solution	Sonication at room temperature in air or under nitrogen gas with 20 kHz US horn having 3 mm diameter micro tip placed between organic and aqueous phases. Sonication time ranged between 30 and 60 s, at 14 W.cm^−2^. Ice bath was used for temperature control	Mean size of 8 ± 3 μm	[136]
Keratin-based microcapsules as potential biocarrier	Emulsification of keratose microcapsules	Sonication of an aqueous-organic mixture of keratose solution and toluene at 20 kHz and 150 W at room temperature for 3 min with stainless steel ultrasonic probe	Microcapsules of diameter 0.5 to 4 μm	[137]
Fructose 1,6-bisphosphate dicalcium (Ca_2_FBP) porous microsphere for osteogenic differentiation	Sonochemically induced (acoustic cavitation effect) crystallisation of Ca_2_FBP	Continuous ultrasonication at 28 kHz and 200 W at intervals of 20 min	Average surface area of 188.5 m^2^ g^−1^ and pore size of 30 nm	[138]
AuNPs as potential cancer treatment agents	US irradiation assisted the Turkevich-Frens method for gold reduction	Sonication at different irradiation power (60 to 210 W) for 1 h at room temperature with ultrasonic homogenizer coupled with titanium sonotrode	Average size of 12.2 to 16.2 nm	[139]
AgNPs anchored reduced graphene oxide nanosheet for selective and sensitive detection of glutathione	Sonochemical co-reduction of AgNO_3_ and grapheme oxide, with resultant condensation of Ag^+^ on reduced graphene oxide	Dispersion of graphene oxide in water and sonication via a standing wave sonication system at 38 kHz and 50 W. Subsequent US induced reduction and deposition of silver with further sonication	Average size of 3 to 8 nm	[140]
Fe_3_O_4_@cellulose nanocrystal stabilised Pickering emulsion for drug delivery	Sonochemical induced fine Pickering emulsion of curcumin and co-precipitation of Fe_3_O_4_@cellulose nanocrystal	Dispersion and synthesis of Fe_3_O_4_@CNC under pulse sonication (10 s on and off) with a probe sonicator at 20 kHz for 2 and 5 min, respectively. Further sonication for 3 min at 60 W to produce fine Pickering emulsion	MNPs agglomeration with an average cluster diameter of 22.998 to 320.668 nm	[141], [142], [143]

## 5 Future outlook and challenges

Ultrasound (US) technology is fairly established in industrial chemical synthesis as well as other processes such as wastewater treatment. In the same way, US is a promising tool for synthesising and modifying nanomaterials widely applied in almost all branches of science and engineering. More work of late could be realised on ultrasonically synthesised nanobiomaterials for various biomedical applications. The examples provided in this review are far from exhaustive yet represent an assorted type of NPs that have been ultrasonically prepared, ranging from organic to inorganic NPs and nanocomposites. Sonically induced cavitation that involves a series of formation, growth and collapse of bubbles in the reaction media, is recognised as the phenomenon that sets off various physical and chemical effects. Nanomaterials have been synthesised top-down by the cavitation forces that break agglomerates and overcome intermolecular forces, and bottom-up through the diffusion and reactions of radical species with solutes within or around the bubble. Reaction time is also significantly reduced through the combined effects of various mechanisms. Under the ultrasonic influence, nanobiomaterials synthesised possess controlled morphology and display improved physicochemical properties. Indeed, a great milestone has been achieved with the use of US for nanobiomaterials synthesis.

Future advances of sonochemical processes are primarily subject to the cost-effectiveness and scalability of the production. US is reproducible, and a linear scale-up is viable [144]. The main criterion for replicating identical results is creating the same energy per volume, while all other configurations should be identical. Equipment sizing can be made based on the energy requirement per weight/volume of processed materials. To determine the economic feasibility, optimising process efficiency requires correlating all relevant parameters. However, there are many controlling parameters among other reaction requirements to fulfil or need to be considered, such as reaction kinetics, and as discussed in this review many require facile techniques for synthesis. To the best of our knowledge, there has not been any published work on the techno-economic assessment and scalability of any sonochemical production of nanomaterials.

Subsequently, we want to highlight two main challenges on this subject: There remain questions unanswered about the actual sonication effect on the reaction dynamics and how could the sonoproduction of nanobiomaterials be implemented on an industrial level to achieve bulk synthesis. To respond to the first challenge, more numerical works at the molecular level is needed to scrutinise the sonication effects, particularly the influence of bubble–bubble interactions on reaction dynamics. Thus far, it has been suggested that low-intensity sonication instead of cavitation effects impact non-radical reactions. This implies the potential use of US in biochemical processes, particularly the immobilisation of temperature-sensitive bioactive compounds and enzymes onto NPs. In response to the second question, Afreen et al. [145] observed that it will be necessary to have a robust protocol to design sonochemical processes. They also outlined some design guidelines on critical sonochemical parameters based on established literature. Appreciably statistical analyses and simulation of critical process parameters will be helpful, and a database of quantitave data from the experimental and numerical sonochemical investigations seem indispensable. In the interim, pragmatic solutions are needed for other concerns such as maintaining the quality of NPs and reducing defects during synthesis.

### CRediT authorship contribution statement

**Sze Shin Low:** Conceptualization, Investigation, Writing – review & editing. **Maxine Yew:** Data curation, Writing – original draft. **Chang Nong Lim:** Methodology, Writing – original draft. **Wai Siong Chai:** Formal analysis, Validation. **Liang Ee Low:** Data curation, Conceptualization. **Sivakumar Manickam:** Writing – review & editing, Project administration. **Beng Ti Tey:** Methodology, Resources. **Pau Loke Show:** .

## Declaration of Competing Interest

The authors declare that they have no known competing financial interests or personal relationships that could have appeared to influence the work reported in this paper.
